# Methylation-associated mutagenesis underlies variation in the mutation spectrum across eukaryotes

**DOI:** 10.1073/pnas.2516368123

**Published:** 2026-03-13

**Authors:** Fabián Ramos-Almodóvar, Ziyue Gao, Benjamin F. Voight, Iain Mathieson

**Affiliations:** ^a^Department of Genetics, Perelman School of Medicine, University of Pennsylvania, Philadelphia, PA 19104; ^b^Genomics and Computational Biology Graduate Group, Perelman School of Medicine, University of Pennsylvania, Philadelphia, PA 19104; ^c^Department of Systems Pharmacology and Translational Therapeutics, Perelman School of Medicine, University of Pennsylvania, Philadelphia, PA 19104; ^d^Institute for Translational Medicine and Therapeutics, Perelman School of Medicine, University of Pennsylvania, Philadelphia, PA 19104

**Keywords:** genetics, mutations, evolution, methylation

## Abstract

Mutation spectra vary across genetic and environmental contexts, leading to differences between and within species. Most research on mutation spectrum has focused on trinucleotide (3-mer) mutation types in mammals, limiting the breadth and depth of variation surveyed. In this study, we use whole-genome resequencing data across 108 eukaryotic species—including mammals, fish, plants, and invertebrates—to characterize pentanucleotide (5-mer) noncoding mutation spectra using a Bayesian approach. Our findings reveal cytosine transition mutability at CpG sites and other sources of variation in the transition/transversion ratio as the main drivers of variation in mutation spectra across eukaryotes. We find that inferred CpG mutation rates almost perfectly predict genomic CpG depletion but are not predicted by genome-wide average CpG methylation levels. Together, our results illustrate the pivotal role of mutagenesis in shaping genome composition across eukaryotes and highlight a gap in knowledge about the mechanisms governing mutation rates.

## Significance

Mutation is the source of all genetic variation. Different types of mutations occur at different rates—the mutation spectrum—which varies across species, shaped by differences in DNA sequence context, damage, replication, and repair. Inferring mutation spectra from 108 eukaryotic species, we demonstrate variation in mutation rates at CpG sites (where a cytosine is followed by a guanine), and in transition/transversion ratio at other sites accounts for most variation in mutation spectra. We also find that CpG mutation rates almost entirely explain genomic CpG content, highlighting the importance of the mutation spectrum in genome evolution.

Mutation is a random process, but it is not uniform. DNA sequence context influences where, what type, and how frequently mutations occur (1, 2). One example is the hypermutability of methylated cytosines at CpG dinucleotides in vertebrate genomes, resulting from spontaneous deamination of 5-methylcytosine to thymine (3, 4). Recognizing the importance of sequence context, recent work has focused on inference of context-specific mutation spectra, which quantify relative mutability based on the flanking nucleotides of a mutated base (5–9). Most studies of mutation spectra have focused on trinucleotide (3-mer) contexts, where the mutation of interest is analyzed along with one adjacent nucleotide on each side. Consideration of the trinucleotide context has facilitated the identification of mutational signatures in cancer genomes as well as characterization of heterogeneity in polymorphism spectrum across human populations (5–7, 10–13). However, extending the analysis to longer sequence contexts provides a higher-resolution view and better captures differences in mutation processes (9, 14, 15). We recently developed *Baymer* (8), a Bayesian hierarchical tree approach that facilitates accurate and robust inference of mutation spectra from polymorphism data. The number of events observed in polymorphism data is much larger (~100 M) relative to de novo (~0.1 M) datasets (16), justifying the use of polymorphism data to infer the mutational spectrum, at least in noncoding regions where the effect of selection is expected to be minimal and not context-specific.

Mutation rates differ across species due to genetic and environmental factors, yet the evolutionary forces shaping context-specific mutation rates remain largely unexplored outside of humans, primates, and a few vertebrates (6, 17–19). With the expansion of large-scale sequencing initiatives and conservation genomics efforts, population-level polymorphism data are now available for diverse eukaryotic taxa beyond mammals (20–22). These resources provide an opportunity to investigate mutation spectra across a wider phylogenetic range. For example, while cytosine methylation is almost exclusive to CpG contexts in vertebrates, it is found extensively in non-CpG contexts in plants and other species where we expect to discover distinct mutational spectra. To this end, we leverage whole-genome resequencing data from 108 eukaryotic species, including mammals, birds, fish, plants, and invertebrates, to characterize variation in mutation spectra in extended pentanucleotide (5-mer) sequence context. Using *Baymer* (8), we infer context-dependent mutation rates from noncoding polymorphisms and assess the biological and evolutionary factors driving mutation spectrum variation.

## Results

### A Catalog of Polymorphisms in the Noncoding Genomes of 108 Eukaryotic Species.

**Fig. 1.**
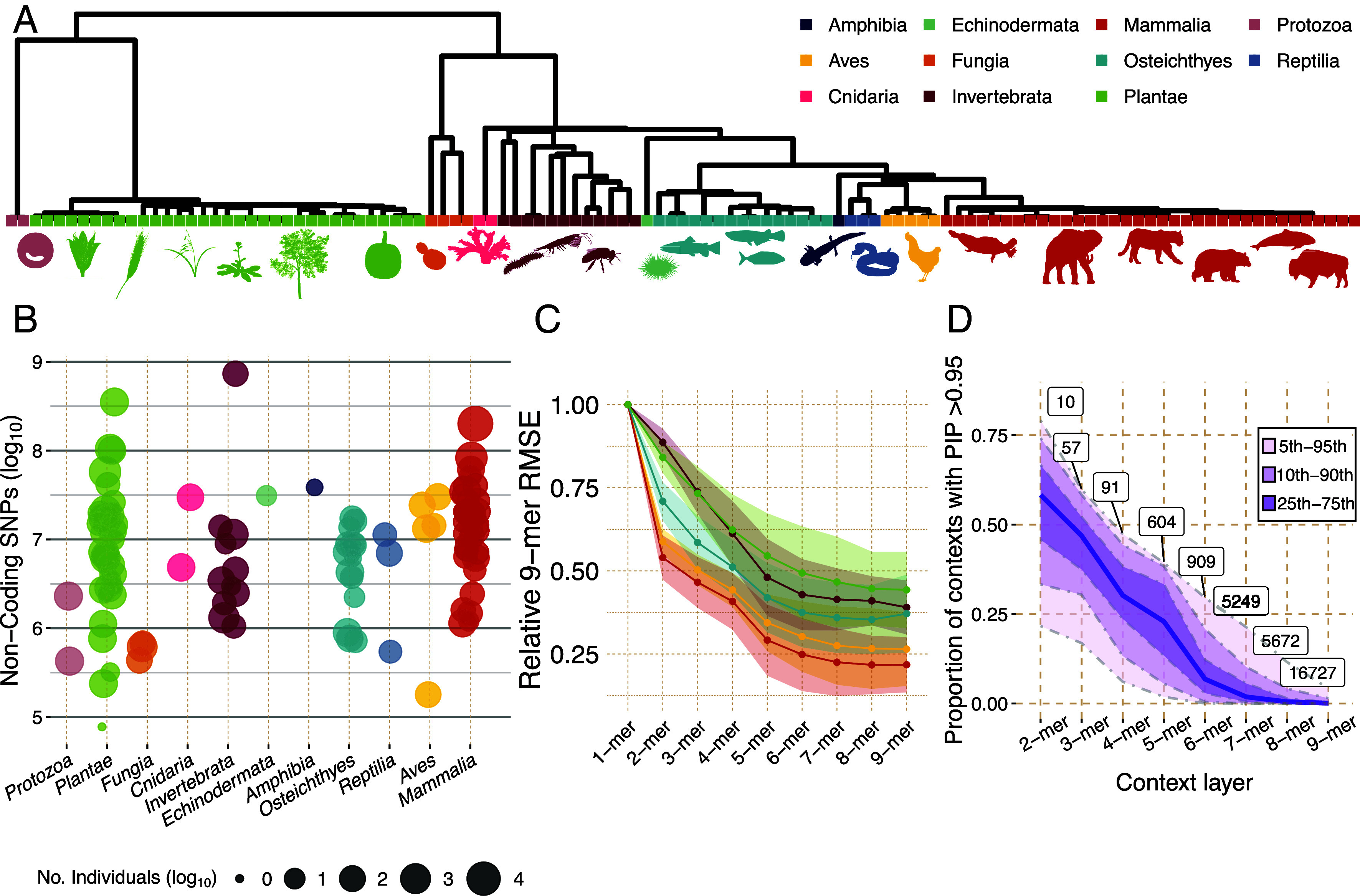
Data summary. (*A*) Phylogenetic tree of collected species, generated with TimeTree, with assigned clades labeled at tips. (*B*) Filtered SNP count (log_10_) per species is shown as individual points, colored by clade and sized by number of individuals (log_10_) in polymorphism dataset. (*C*) RMSE between a k-mer mutation model trained on odd-numbered basepairs vs. a 9-mer mutation model trained on even-numbered basepairs. Points show mean RMSE across species grouped by clade (only clades with n >= 5 are shown). Shaded areas show one SD from the mean. (*D*) Proportions of contexts with posterior inclusion probability (PIP) greater than 0.95 in each context layer, for every species. The dark line represents the median proportion across species; shaded areas represent labeled quantiles. Number of contexts in 95th percentile is labeled for each layer.

The availability of population polymorphism data in diverse species allows us to study the evolution of mutational mechanisms across many previously unexplored lineages (20, 21). We collected publicly available polymorphism data from whole-genome shotgun sequencing for 113 eukaryotic species (23–122), including 35 mammals, 5 birds, 15 fish, 33 plants, and 12 invertebrates (Fig. 1*A* and Dataset S1). To reduce issues related to genome assembly quality, variant callability, and the effects of selection on coding regions, we masked repetitive elements, low complexity regions, and exons from each species assembly, with the resulting regions subsequently referred to as the accessible noncoding genome. This resulted in polymorphism datasets with 10^5^ to 10^9^ SNPs per species (Fig. 1*B*).

### Baymer Captures Variation in Mutation Spectra across Species.

We used *Baymer* (8) to infer sequence context windows (up to 4 flanking nucleotides, i.e., “9-mers”) separately in each of the 113 species. *Baymer* infers the probability of polymorphism–an approximation to the relative mutation rate of each mutation type–by fitting a hierarchical Bayesian model to the number of observed polymorphisms and the corresponding frequency of the mutable context. A spike-and-slab prior allows contexts to have zero effect on mutation rate, preventing overfitting and allowing the model to adapt to species with very different numbers of observations. We use the default prior and MCMC settings as described in Adams et al. (8).

To evaluate the performance of our mutation models within species, we computed the root mean squared error (RMSE) between the mutation models generated from odd- and even-basepairs of the accessible noncoding regions (*SI Appendix,* Fig. S1). A higher RMSE suggests higher uncertainty in estimated rates due to technical noise or data sparsity. There is a large drop in RMSE between 1-mer and 2-mer models for mammals and birds due to the inclusion of CpG contexts. For all species, RMSE decreases for larger contexts, but the decrease is substantially slower above 5-mer contexts (Fig. 1*C*). This reflects the observation that the average posterior inclusion probability (PIP)–the proportion of contexts with a nonzero effect–drops from 25% at the 5-mer level to 2% at the 7-mer level (Fig. 1*D*). We noted five species with near zero PIPs for every mutation context across every context layer (*SI Appendix,* Fig. S3)–a pattern expected in random noise or sparse data that are not informative of mutational processes. We therefore excluded these five species from further analysis and focused on 5-mer contexts for downstream analyses in the 108 remaining species.

### Cytosine Transitions at Methylation Target Sites Explain Most of the Variation in Mutation Spectra.

**Fig. 2.**
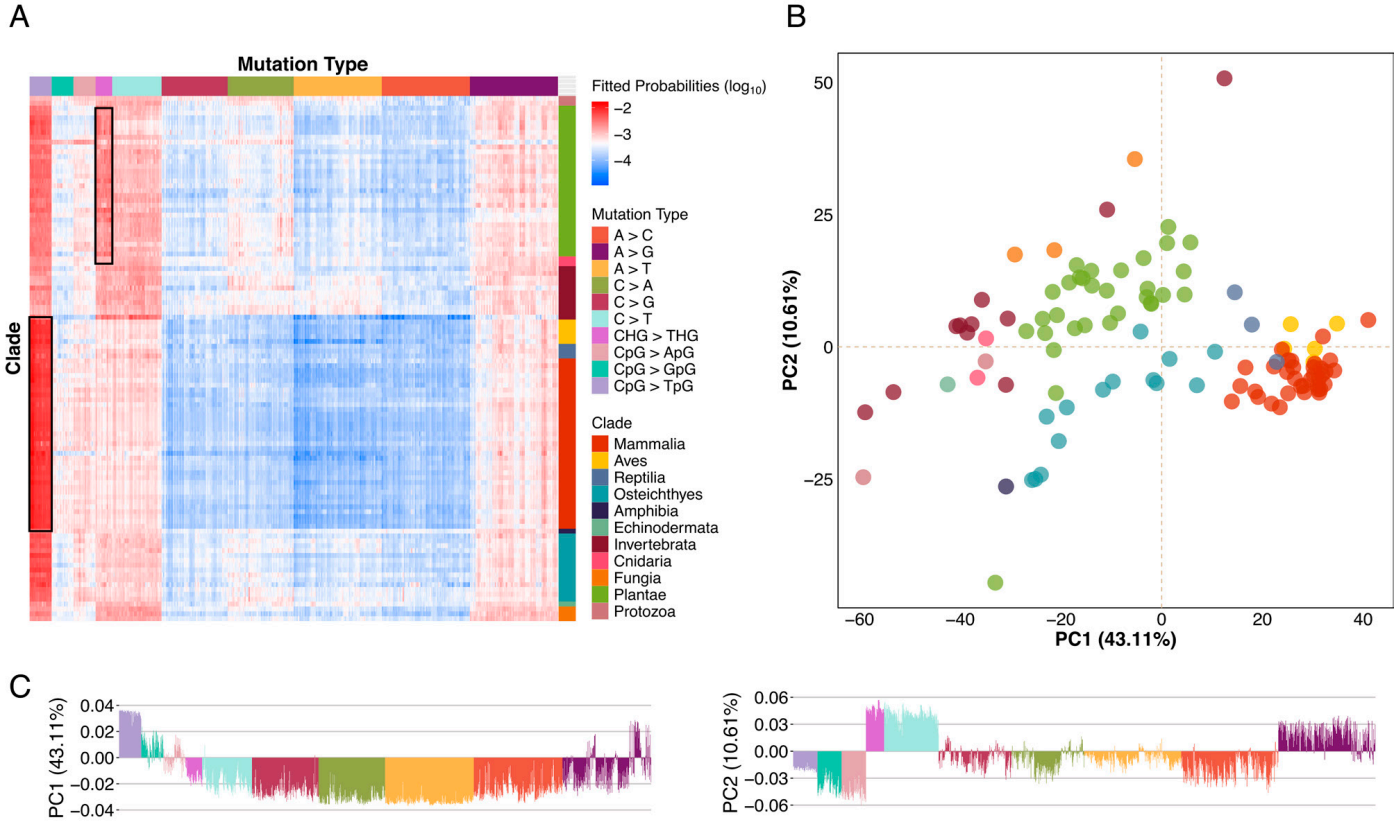
PCA of scaled fitted 5-mer polymorphism probabilities. (*A*) Heatmap of raw 5-mer context fitted probabilities; CpG>T and CHG>THG mutations are highlighted in boxes for tetrapods and plants, respectively. (*B*) First two principal components from PCA on scaled polymorphism probabilities. Points show each species, colored by clade. (*C*) First two principal component loadings, showing individual bars for each mutation type, colored by mutation type.

To investigate the variation of context-specific mutation rates in eukaryotes, we first performed principal component analysis (PCA) on the unscaled fitted 5-mer context-specific mutation spectra (Fig. 2*A*), noting that PCA applied to 7-mer (but not 3-mer) contexts produced qualitatively similar results (*SI Appendix,* Fig. S4). We found that the first two principal components cluster mutation spectra by clade and capture 84% (PC1: 79.5%, PC2: 4.56%) of the variance in 5-mer mutation spectra across eukaryotes (*SI Appendix,* Fig. S5*A*). The first and second principal components are characterized by variation in NNCGN > NNTGN and NNCHG > NNTHG mutation rates, respectively (where N is any nucleotide and H is any nucleotide other than guanine; *SI Appendix,* Fig. S5*B*). We ruled out saturation of CpG sites due to sequencing of large sample sizes as the source of the captured variation by computing the proportion of CpG sites that are polymorphic for each species (*SI Appendix,* Fig. S6).

While the PCA on unscaled mutation spectra reveals variation in absolute contributions to the mutation spectra, it can obscure relative differences due to the disproportionate contribution CpG transitions to the PCA loadings. To assess relative differences in mutation spectra, we performed PCA on scaled 5-mer mutation spectra, where the rates of each mutation type were standardized to have unit variance across species. When examining this relative variation, we found that the first two principal components capture 53.72% (PC1: 43.11%, PC2: 10.61%) of the variance in 5-mer mutation spectra across eukaryotes, and the species still cluster by clade and form a similar phylogenetic cline (Fig. 2*B*). PC1 of the scaled mutation spectra is characterized by CpG mutations vs. other mutation types and PC2 by variation in non-CpG transitions vs. other mutation types (Fig. 2*C*). Mammals, birds, and reptiles have the highest weights in PC1, consistent with higher relative CpG mutation rates. The correlation between PCA results and context-specificity of cytosine methylation indicates that most of the variation in context-specific mutation rates across eukaryotes is driven by variation in the rate of cytosine transitions at CpG sites. These results underscore transitions at cytosine methylation target sites as the main driver of context-specific mutation rate variation across eukaryotes.

### CpG Transition Rates Predict Genomic CpG Depletion but Are Not Predicted by Methylation Levels.

**Fig. 3.**
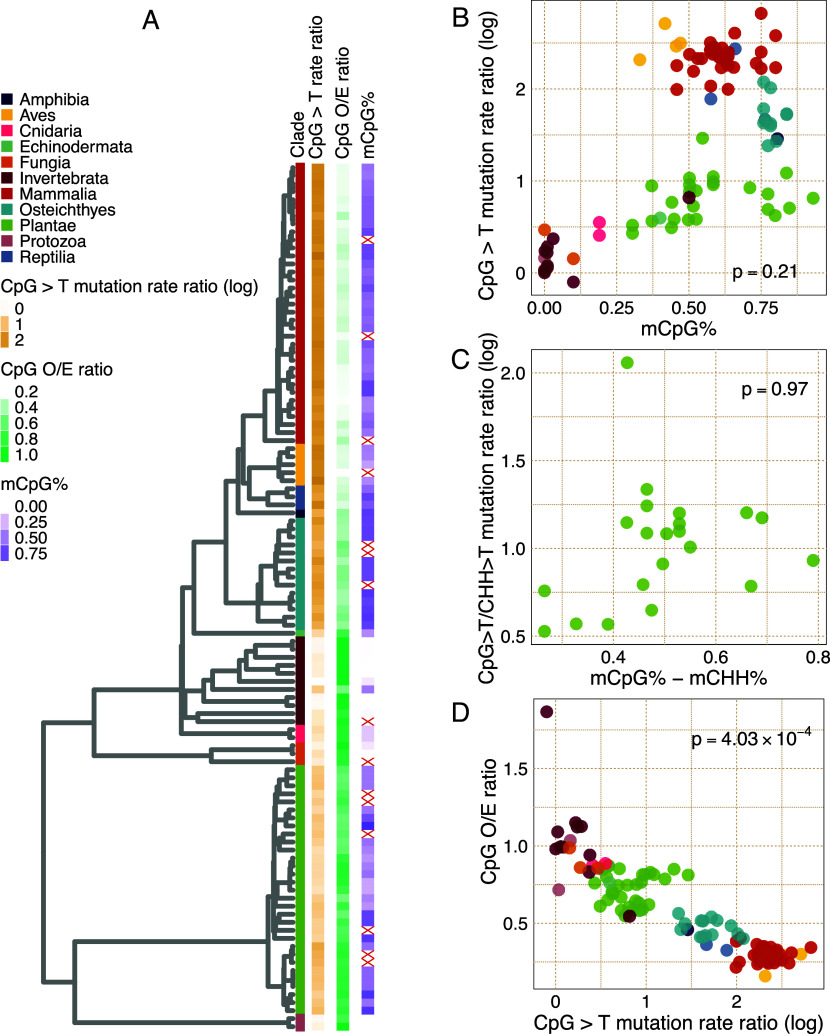
CpG methylation level, mutation rate, and genome composition. (*A*) Phylogenetic tree with CpG-related data shown at tips (red crosses denote missing values). (*B*) CpG>T mutation rate ratio vs. genome-wide average CpG methylation. (*C*) Same as (*B*) but restricted to plants and removing the effect of CHG sites on CpG mutation rate ratio and methylation level. (*D*) O/E CpG noncoding genome composition vs. CpG>T mutation rate ratio. (*B*–*D*) *P*-values correspond to phylogenetic regression results.

We used the ratio of CpG>T and CpH>T polymorphism probabilities to measure the change in mutation rate due to the CpG context and refer to this as the CpG>T mutation rate ratio. Consistent with the mutagenic effect of methylation, this ratio is largest (5- to 20-fold) in vertebrates, which have high levels of CpG methylation, lower (1.5 to 4.5-fold) in plants and less than 1.5-fold in species without substantial methylation (Fig. 3*A*).

However, a closer analysis based on genome-wide CpG methylation levels derived from whole-genome bisulfite sequencing (WGBS) (24, 123–133) reveals a more complicated pattern. Phylogenetic linear regression of CpG>T mutation rate ratio on average CpG methylation is not significant (*P* = 0.21; Fig. 3*B*). This lack of relationship is clearly visible among vertebrates, where fish have high methylation levels but low mutation rate ratios, and birds vice versa. We note that the methylation data we collected is derived from somatic tissues. For seven vertebrates where germline methylation data were available (134–139), we find a Spearman correlation of 0.595 between somatic and germline methylation (*SI Appendix,* Fig. S7*A*) and a relationship that is consistent with the somatic mutation data (i.e., higher methylation but lower CpG mutation in fish relative to mammals; *SI Appendix,* Fig. S7*B*). Thus, presence of CpG methylation entails higher CpG>T mutation rate, but genome average CpG methylation levels as currently measured do not predict the magnitude of the rate.

In addition to CpG methylation, many plants have methylation in CHG and CHH contexts. We therefore corrected our analysis of CpG mutability by taking CHH contexts as the reference, regressing the CpG>T/CHH>T mutation rate ratio on the difference between CpG and CHH genome-wide average methylation levels in plants (Fig. 3*C*) and observed consistent results (phylogenetic regression *P* = 0.97).

We next evaluated the effect of the CpG>T mutation rate ratio on CpG content of the accessible noncoding genome, as measured by the ratio of observed CpG fraction among all dinucleotides and the expected CpG fraction based on GC content [CpG Observed/Expected (O/E) ratio]. We found that the CpG>T mutation rate ratio strongly predicts the magnitude of CpG depletion (phylogenetic regression *P* = 4.03 × 10^−4^) (Fig. 3*D*), with no depletion in species without elevated CpG>T mutation rate ratio; within-clade analysis is reported in *SI Appendix,* Table S1. This aligns with the expectation of lower equilibrium genome representation of highly mutable contexts and suggests that CpG content in the noncoding genome is largely determined by the mutation spectrum with minimal influence of selective constraint on CpG content. The one outlier is the honeybee *Apis mellifera*, which has previously been noted to have an exceptionally high genomic CpG content (140). To provide additional evidence, we examined changes along terminal branches, which offer a cleaner test of this relationship by focusing on recent evolutionary changes. We inferred the CpG O/E ratio and relative mutation rate at the internal nodes of the tree using fastAnc in R and quantified the changes along terminal branches (*SI Appendix,* Fig S8). We observed a significant overall negative correlation between shifts in CpG O/E ratio and relative mutation rate, consistent with the expectation that species experiencing recent increases in CpG mutation rates are moving toward lower CpG composition (and vice versa). Although this correlation reaches significance only in vertebrates and invertebrates, driven predominantly by honeybee and krill, the negative trend is consistent across other clades (*SI Appendix,* Table S2).

### In Plants, CHG Transition Rates Predict CHG Depletion but Are Not Predicted by Methylation Levels.

**Fig. 4.**
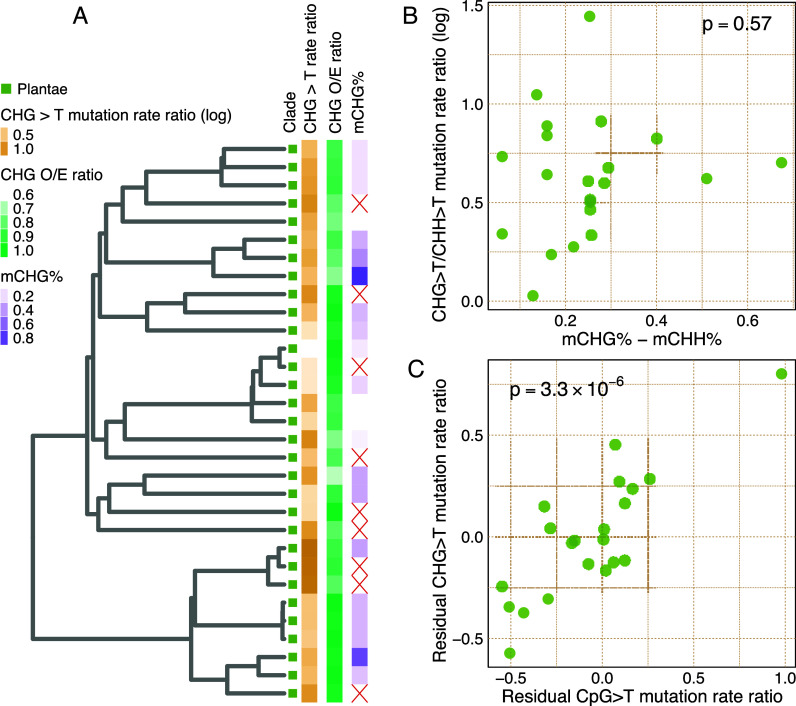
Plant CHG methylation and mutation rate. (*A*) Phylogenetic tree with CHG data shown on tips (red crosses denote missing values). (*B*) CHG>T mutation rate ratio vs. genome-wide average CHG methylation. (*C*) Plant CpG and CHG mutation rate ratio residuals from phylogenetic regressions of CpG>T mutation rate ratio on CpG methylation vs. those from CHG>T mutation rate ratio regressed on CHG methylation. Plant species are shown as individual points (Pearson’s r = 0.829).

To test the relationship between CHG methylation, mutation rate, and genome composition in plants, we extracted genome-wide average CHG methylation levels from WGBS (123) and computed the CHG>T/CHH>T mutation rate ratio and the CHG observed-to-expected (O/E) ratio for each species. Analogous to the findings for CpG methylation across eukaryotes, we found that the CHG>T mutation rate ratio is not predicted by CHG methylation (phylogenetic regression *P* = 0.57, Fig. 4*B*) but does predict the CHG O/E ratio (phylogenetic regression *P* = 6.75 × 10^−7^, *SI Appendix,* Fig. S9). To investigate whether the same or different factors underlie variation in mutability of CpG and CHG contexts, we regressed our species’ mutation rate ratios on their methylation levels separately for CpG and CHG contexts and found that the residuals were highly correlated (Pearson’s coefficient = 0.829; *P* = 3.3 × 10^−6^) (Fig. 4*C*) consistent with shared mechanisms underlying residual variation in both.

### Mutation Signature Analysis Confirms Transitions at Methylation Target Sites as the Main Driver of Mutation Spectrum Variation in Eukaryotes.

**Fig. 5.**
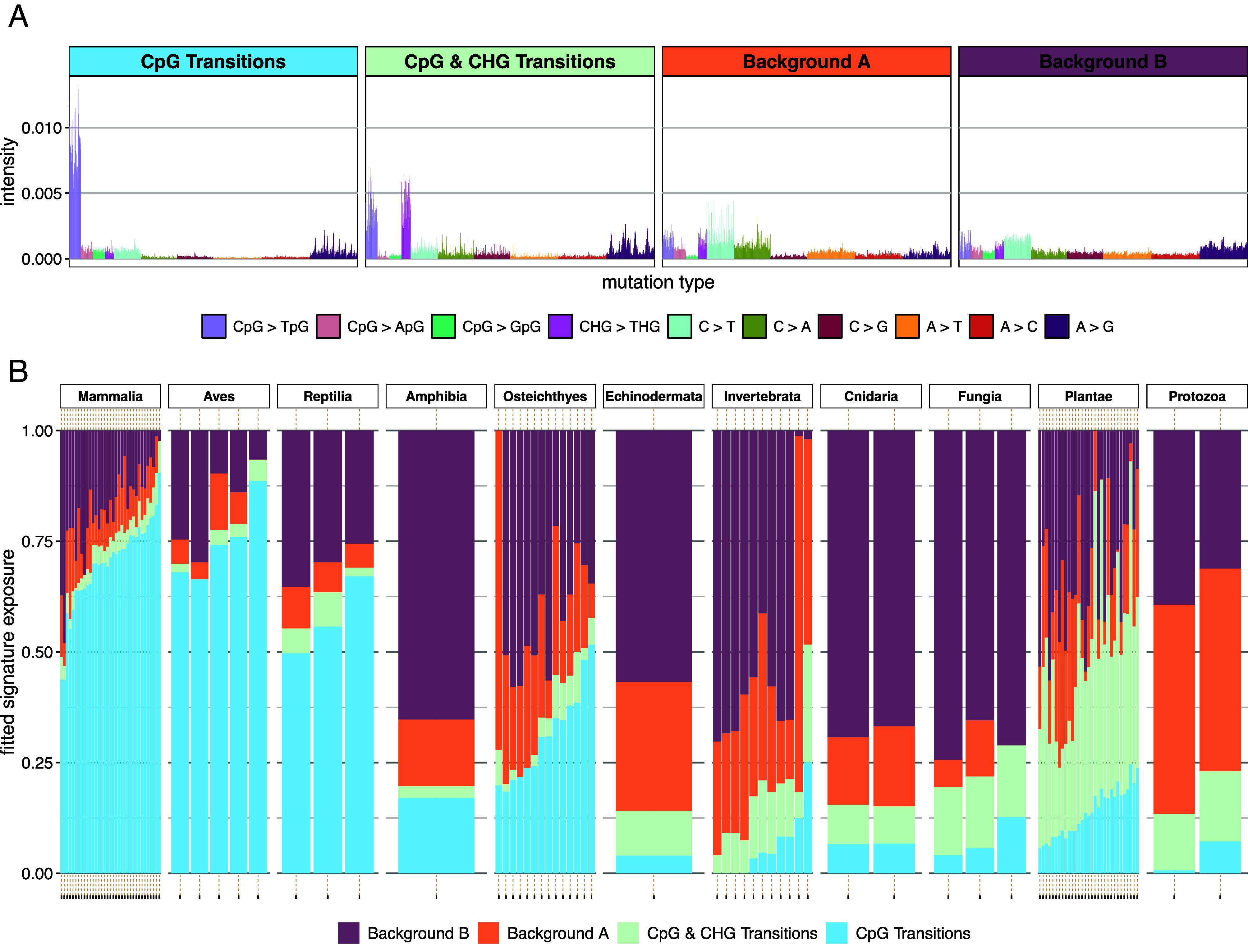
Mutational signature analysis in 5-mer polymorphism spectra. (*A*) Extracted signature mutation spectra. (*B*) Estimated contributions from each inferred signature to each species’ polymorphism counts.

To corroborate our findings from Baymer-inferred context-specific unscaled mutation rates, we used SigFit (141) to model the observed 5-mer polymorphism spectrum in each species as a linear combination of *k* mutational signatures weighted by differential exposures in each species. Following Beichman et al. (142), we computed the cosine similarity between the observed and reconstructed mutation spectrum vectors to evaluate model fit to the data. After analyzing the reconstruction performance for *k* = 2 to 10, we determined that four mutational signatures had optimal performance and interpretability (*SI Appendix,* Fig. S10). These four signatures reflect a CpG transition signature, a CpG and CHG transition signature, and two background signatures (Fig. 5*A*). Consistent with our previous observations, the CpG transition signature was most active in mammals, birds, and reptiles while the signature of CpG and CHG methylation was most active but variable in plants (Fig. 5*B*); the two background signatures are present in all clades.

## Discussion

Our study provides a comprehensive analysis of context-specific mutation spectra across 108 eukaryotic species, leveraging a phylogenetically diverse collection of polymorphism data from whole-genome sequencing. We find that variation in the mutation spectrum largely reflects variation in the rates of CpG>T mutations and in transitions at non-CpG sites (Fig. 2). In plants, where methylation in CHG contexts is common, extending to 5-mer contexts allows us to identify a substantial role for CHG>T mutations in shaping the mutation spectrum (Fig. 2*A* and *SI Appendix,* Fig. S5).

While our findings reinforce the predominant role of transitions at methylation target sites on mutation spectrum variation across eukaryotic species, they also suggest the possibility that methylation is not the only factor at play, as genome-wide average methylation levels do not predict mutation rates at these sites across species after correcting for phylogenetic nonindependence. Therefore, while methylation is necessary for high transition rates at CpG and CHG sites, the variation in mutation rates may be modulated by other unknown genetic and environmental factors that differ across species and modify rates of deamination, replication error, or repair. One limitation of our current analysis is that it is based on somatic methylation data which might imperfectly reflect methylation patterns in the germline. Consequently, a poor correlation between methylation levels and mutation rates may be observed even if the former strongly determines the latter in the germline. If that is the case, our observations could reflect uncoupling between somatic and germline methylation levels across species. The relationship between mutation and methylation should be reanalyzed when germline methylation data are available for a broader range of eukaryotic species. In contrast, we found that genomic CpG and CHG depletion is highly correlated with cytosine transition rates at CpG and CHG sites, suggesting that these aspects of genomic composition are largely determined by mutational pressures rather than selection, biased gene conversion, or other forces.

In summary, this study advances our understanding of mutation spectrum variation across eukaryotes by integrating 5-mer context mutation spectrum models over a wide range of species. We show that polymorphism data serve as a reliable proxy for mutation data, although absolute mutation rate cannot be assessed and fine-scale variation may be missed due to data artifacts or the lack of polarization of ancestral alleles. Future work should aim to identify genetic or environmental correlates of CpG and CHG transition rates after accounting for methylation, investigate variation across genomic compartments, and sample more broadly from clades with extreme mutational spectra or genome compositions.

## Methods

### Data Collection.

#### Polymorphism data.

We obtained species polymorphism datasets, in the form of .vcf files, from public repositories or directly from authors. We carried out this process by searching through the literature and limiting our selected species to those with polymorphisms called from whole-genome sequencing data, with at least 2× coverage and five individuals (excluding humans, sample size: median = 81; mean = 259). We also required that the reference genomes used for variant calling had coding region annotations. The full list of species, along with data sources, can be found in Dataset S1.

For *Ornithorhynchus anatinus* (Platypus), we aligned 49 individual genomes (in FASTQ format) and called variants using DeepVariant (143) with its standard protocol.

#### Reference genome assembly.

Corresponding reference assemblies for each species were downloaded from NCBI or publication repository, along with coding and RepeatMasker annotations. Dataset S1 contains list of sources for polymorphism data and reference genome assembly ID.

### Data Preparation.

#### Noncoding genome.

For each species, we limited the analysis to the accessible noncoding genome by masking out exons, repetitive elements, and low complexity regions. For coding sequences, we extracted genome coordinates (in the form of .bed files) for exons from coding sequence annotation files (downloaded from NCBI or publication repository in the form of .gtf or .gff files) generated from RNA sequencing, for the vast majority of our species (Dataset S1). Repetitive element coordinates were extracted from RepeatMasker annotations. When RepeatMasker annotation was not available, we used *RepeatMasker-4.1.5* with default parameters and specified clade repeat libraries to generate repetitive element annotations. Low-complexity DNA regions were masked using the NCBI tool, *DustMasker-1.0.0* (with -window = 32; default is 64). Our final genomic region for analysis was defined using *bedtools2-2.30.0 getfasta* function by excluding genome coordinates that overlapped exonic, repetitive, and low-complexity coordinates from the above.

#### Polymorphism data.

We retained only single-nucleotide polymorphisms (SNPs) from the variant call format (vcf) files with complete 9-mer contexts in the accessible noncoding genome (defined above). Multiallelic sites were treated as independent mutations at the same site. Since the ancestral genome assembly is unavailable for most species, we used reference alleles for polarizing the polymorphisms, assuming the reference allele is the ancestral allele and nonreference allele the mutated allele. Reverse complementary mutation types were combined. To mitigate concerns of high error rate in singletons, we restricted our analysis to SNPs present in at least two individuals, when individual genotypes were available; otherwise, we removed singletons based on nonreference allele count. Because our polymorphism data derive from studies with different sequencing and variant calling strategies, we checked that the PCs of the mutation spectrum were uncorrelated with technical features including SNP count and reference genome N50 (*SI Appendix,* Fig. S8).

### Calculation of CpG and CHG O/E Ratio.

The observed-overexpected CpG content ratio (CpG_O/E_) was calculated as

[1] $${\text{CpG}}_{O/E}=\frac{[\mathrm{CpG}{]}_{f}}{[C{]}_{f}[G{]}_{f}},$$

where the observed CpG frequency, $[CpG{]}_{f}$, was calculated as the fraction of dinucleotide counts that are CpGs and the expected CpG frequency the product of the observed cytosine and guanine single-nucleotide frequencies, $[C{]}_{f}$ and $[G{]}_{f}$, in the accessible noncoding genome.

The observed-overexpected CHG content ratio (CHG_O/E_) was calculated similarly as

[2] $${\mathrm{CHG}}_{O/E}=\frac{[\mathrm{CHG}{]}_{f}}{[C{]}_{f}[H{]}_{f}[G{]}_{f}},$$

where the observed CHG (where H is any nucleotide other than guanine) frequency, $[\mathrm{CHG}{]}_{f}$, was calculated as the fraction of trinucleotide counts that are CHGs and the expected frequency the product of the observed frequencies of cytosine, nonguanine, and guanine single-nucleotide, $[C{]}_{f}$, $[H{]}_{f}$, and $[G{]}_{f}$, in the accessible noncoding genome.

### Methylation Data Collection.

#### Cytosine methylation data.

We collected averaged genome-wide cytosine methylation levels at CpG sites in each species and at CHG sites in plants from multiple sources (each species’ methylation state and source is indicated in Dataset S2) from WGBS studies (123, 124). Additionally, we collected averaged genome-wide cytosine methylation levels at CpG sites from testes WGBS for seven vertebrate species (134–139) (listed in Dataset S2).

### Context-Specific Mutation Probability Models Using Baymer.

To construct our mutation probability models, we used Baymer (8), a Bayesian hierarchical tree model approach that estimates context-specific mutation probabilities for increasing context layers, iteratively. Baymer generates regularized mutation probability estimates and provides a measure of uncertainty for each multiplicative shift in mutation probability leading up to the final k-mer mutation context. The context-specific mutation rates for any given context window are calculated as the product of the multiplicative shifts leading to the final mutation context. To generate context-specific mutation probability models from polymorphism data, Baymer requires a table with DNA context counts and polymorphic sites for the largest context size desired for the models (in our case, 9-mers). For each species, we first generated a table of 9-mer context counts within the accessible noncoding genome (defined above). Then, we identified and counted polymorphic sites with complete 9-mer contexts in the same regions. Lower context size counts (e.g., 5-mers) were derived from the 9-mer context size counts.

### Assessing Robustness of the Mutation Model.

We applied Baymer to these inputs to generate three models, each using one of three different partitions of the noncoding genome, which we refer to as ALL, ODD, and EVEN. The ALL model consisted of every context and polymorphism in the accessible noncoding genome, while the ODD and EVEN models were only generated using odd or even genome coordinates in the accessible noncoding genome, respectively. The ODD and EVEN models were generated for cross-validation of model performance in each species. We assessed within-species model robustness by computing the RMS error (RMSE) between the ODD and EVEN polymorphism probability estimates; calculated as

[3] $${\text{RMSE}}_{\text{Odd}/\text{Even}}=\frac{1}{K}\sqrt{\sum_{i=1}^{K}(\text{log}\left(O_{i}\right)-\log\left(E_{i}\right){)}^{2}},$$

Where **O** and **E** are the fitted context-specific rate vectors, of length *K*, from the ODD and EVEN models, respectively. *SI Appendix,* Fig. S1 shows the RMSE_Odd/Even_ for the 5-mer mutation models. Fig. 1*C* shows the RMSE between 9-mer EVEN mutation models vs. k-mer (k = 1 to 9) ODD mutation models, normalized by the k = 1 RMSE.

### Mutation Rate Ratios.

Our mutation models infer content-specific relative mutation rates rather than absolute mutation rates. For this reason, we base our mutability analyses on relative differences in context-specific mutation rates, which we refer to as “mutation rate ratios.” We estimated these ratios using 5-mer context-specific mutation rates.

CpG>T mutation rate ratio was defined for each species as the ratio of the average C>T mutation rate in all 5-mer CpG contexts over that of CpH contexts (H = A,C,T):

[4] $$\mathrm{CpG}>T\;\mathrm{Mutation}\;\mathrm{Rate}\;\mathrm{Ratio}=\frac{\frac{1}{K}\sum_{i=1}^{K}[\mathrm{NNCGN}>\mathrm{NNTGN}]}{\frac{1}{N}\sum_{j=1}^{N}[\mathrm{NNCHN}>\mathrm{NNTHN}]},$$

where the summation is across all 5-mer contexts matching the specified patterns.

We note that plant species also experience extensive cytosine methylation at non-CpG contexts. Because of this, the CpG>T mutation rate ratio above may be a biased representation of the relative context-specific CpG>T mutation rate in these plants. To account for this, we defined the CpG>T/CHH>T mutation rate ratio for each plant species as the ratio of the average C>T mutation rate in CpG contexts over that of CHH contexts, removing impacts of methylation in CHG contexts:

[5] $$\frac{\mathrm{CpG}>T}{\mathrm{CHH}>T}\mathrm{Mutation}\;\mathrm{Rate}\;\mathrm{Ratio}=\frac{\frac{1}{K}\sum_{i=1}^{K}[\mathrm{NNCGN}>\mathrm{NNTGN}]}{\frac{1}{N}\sum_{j=1}^{N}[\mathrm{NNCHH}>\mathrm{NNTHH}]}.$$

To analyze the relationship between CHG transition rates, methylation, and genome composition in plants, we defined the CHG>T/CHH>T mutation rate ratio for each plant species as the ratio of the average C>T mutation rate in 5-mer CHG contexts over that of CHH contexts:

[6] $$\frac{\mathrm{CHG}>T}{\mathrm{CHH}>T}\mathrm{Mutation}\;\mathrm{Rate}\;\mathrm{Ratio}=\frac{\frac{1}{K}\sum_{i=1}^{K}[\mathrm{NNCHG}>\mathrm{NNTHG}]}{\frac{1}{N}\sum_{j=1}^{N}[\mathrm{NNCHH}>\mathrm{NNTHH}]}.$$

### Phylogenetic Tree.

Our phylogenetic tree (in Newick file format) was constructed using estimated divergence times from TimeTree (timetree.org). Dataset S1 contains the list of species names used for TimeTree.

### PCA on Raw and Scaled Fitted Mutation Probabilities.

We performed PCA on the raw 3-mer, 5-mer, and 7-mer context size fitted mutation probability models, separately. We scaled the fitted mutation probabilities for each species so that they added up to one and used the prcomp(scale = FALSE, center = TRUE) R (v4.2) function to perform PCA based on single-value decomposition for supplementary figures. For the main text in Fig. 2, we performed PCA on scaled 5-mer context mutation probabilities, by standardizing each mutation type to have mean = 0 and SD = 1 across species before PCA; prcomp(scale = TRUE, center = TRUE).

### Phylogenetic Linear Regressions.

To perform analysis accounting for the phylogenic tree structure given the species included in our study, we used the *phylolm-2.6.2* R package phylolm() (144) function with Pagel’s lambda as a phylogenetic scaling factor and 1000 bootstraps for our regression models. The phylolm() function is an implementation of the phylogenetic generalized least squares regression model:

[7] $$\hat{\beta }=(X^{T}V(\lambda {)}^{-1}X{)}^{-1}X^{T}V(\lambda {)}^{-1}y,$$

where for *n* species and *k* independent variables, ***y*** (*n* × 1) is the continuous outcome vector, β (*n* × 1) is the vector of coefficients for the variable matrix ***X*** (*n* × *k*), and ***V*** (*n* × *n*) is the variance–covariance matrix based on the phylogenetic relatedness between the species. Pagel’s lambda is then used to transform ***V*** into a new matrix, ***V***(λ) (*n* × *n*), by scaling the off-diagonal elements of ***V*** using the scalar λ, which is an estimate of phylogenetic signal for the regressed trait. λ ranges between 0 and 1, where 1 indicates that the covariance structure is exactly given by the phylogenetic relatedness structure, and 0 indicates no phylogenetic signal for the regressed trait. We report two-tailed *P*-values.

### Mutational Signature Analysis with SigFit.

We extracted mutational signatures from the observed 5-mer polymorphism spectra using a nonnegative matrix factorization approach, implemented in the *SigFit-2.2* (141) R package. SigFit uses a Bayesian multinomial model to fit specified and/or inferred mutational signatures to observed mutation spectra, an approach equivalent to nonnegative matrix factorization. For **G** genomes and **M** mutation contexts, SigFit takes as input a mutation counts matrix (**G** by **M**) and a mutation opportunities matrix (**G** by **M**); the mutation opportunities matrix contains the number of mutable contexts for each given 5-mer mutation type in the species’ genome. For our SigFit analysis, we used similar mutation and context counts as inputs for Baymer inference. We used the extract_signatures() function from SigFit, with iter = 30000 and nsignatures = k (for k = 2,3, …, 10). For each SigFit model, we reconstructed the mutation spectra using the fitted mutational signatures and their estimated activity values in each species. We then computed the cosine similarity between the observed and reconstructed 5-mer mutation spectra vectors to measure each model’s performance in each species.

## Supplementary Material

Appendix 01 (PDF)

Dataset S01 (XLSX)

Dataset S02 (XLSX)

## Data Availability

Code to reproduce the analysis in this paper is available at https://github.com/framos99/MutationSpectraEvolutionAcrossEukaryotes-Project (145) See *SI Appendix*, Table S1. Fitted mutation spectra for each species are available at https://doi.org/10.5281/zenodo.15464759 (146). Original sources for data are given in Dataset S1. All other data are included in the manuscript and/or supporting information.
